# Rho GTPase-activating protein 17 (ARHGAP17) as additional autoimmune target in ARHGAP26-IgG/anti-Ca autoantibody-associated autoimmune encephalitis

**DOI:** 10.1007/s00415-022-11417-z

**Published:** 2022-11-04

**Authors:** Sven Jarius, Jürgen Haas, Brigitte Wildemann

**Affiliations:** Molecular Neuroimmunology Group, Department of Neurology, University of Heidelberg, Heidelberg, Germany

**Keywords:** Rho GTPase-activating protein 26 (ARHGAP26), Rho GTPase-activating protein 17 (ARHGAP17), RhoGAP interacting with CIP4 homologs protein 1 (RICH-1), Nadrin, Rho GTPase-activating protein 10 (ARHGAP10, GRAF2), GTPase-activating protein 2 (ARHGAP2, N-chimerin, chimerin-1), Autoantibody, Immunoglobulin G (IgG), Autoimmune encephalitis, Cerebellar ataxia, Limbic encephalitis, Polyneuropathy, Cognitive decline, GTPase Regulator Associated with Focal Adhesion Kinase (GRAF), Oligophrenin-like protein 1 (OPHN1L)

Dear Sirs,

In 2010, we described a novel serum reactivity targeting the Rho GTPase-activating protein 26 (ARHGAP26; also termed GTPase Regulator Associated with Focal Adhesion Kinase [GRAF], or oligophrenin-like protein 1 [OPHN1L]) [1]. Immunohistochemically, ARHGAP26-IgG (also termed anti-Ca) is characterized by a distinct, medusa head-like binding pattern on cerebellar tissue sections [2–4], corresponding to staining of Purkinje cell somata and dendrites. ARHGAP26-IgG/anti-Ca was first described in patients with autoimmune cerebellar ataxia (ACA) [1]. However, the spectrum of clinical syndromes associated with this new reactivity was later shown by us and others to also include limbic encephalitis, cognitive decline, and polyneuropathy [5–10]. In one-third (8/24) of all cases reported so far, autoimmunity to ARHGAP26 was associated with a definite tumor diagnosis (1 × ovarian; 1 × prostate; 3 × squamous cell; 1 × breast; 1 × monoclonal gammopathy; 1 × gastric adenocarcinoma). In two further cases, radiological findings compatible with lymph node metastases (of occult origin) were present. This makes ARHGAP26-IgG/anti-Ca an intermediate-risk paraneoplastic autoantibody according to current criteria [11]. In one additional case, the syndrome was associated with thymic hyperplasia.

Recently, we demonstrated in a study published in this journal that ARHGAP26-IgG/anti-Ca-positive sera cross-react in the vast majority of cases with ARHGAP10 (GRAF2; not to be mixed up with ARHGAP21, which has been occasionally falsely referred to as ARHGAP10 [12]). ARHGAP10 shares a significant sequence homology with ARHGAP26 and has been shown to be expressed at the RNA level throughout the CNS and at the protein level in the cerebellum in particular [13–15]. Here, we report that IgG from the ARHGAP26-IgG/ARHGAP10-IgG-positive index patient [1], who had presented with acute cerebellar ataxia (with cerebellar atrophy on MRI), diplopia, hyperekplexia, restlessness, anxiety and severe depression, reacts in addition with ARHGAP17 (also termed RhoGAP interacting with CIP4 homologs protein 1 [RICH-1]). Reactivity with ARHGAP17 was found by an in-silico re-evaluation of the original microarray (Protoarray v5.0; Invitrogen) experiment leading to the identification of ARHGAP26 as target antigen in ACA [1]. While ARHGAP26 showed the by far strongest IgG reaction among the > 9000 proteins tested in total in that experiment (signal rank 1; median fluorescence units [FU] at 635 nm: 55,232; median FU of all proteins: 181; *Z*-factor 0.89; *Z*-factor cut-off: 0.4), ARHGAP17 was among the hits as well, albeit with lower signal intensity (signal rank 31; median fluorescence units [FU] at 635 nm: 9296.5; *Z*-factor: 0.89). However, when eliminating those hits from the analysis that were either immunoglobulin-related (representing false-positives due to direct binding of the secondary antibody used to detect bound IgG) or considered non-specific by the manufacturer based on their regular occurrence in healthy control samples, only the signals of ARHGAP26 and coilin ranked higher than that of ARHGAP17. Anti-coilin is a nuclear antigen antibody rarely associated with connective tissue disorders (CTD) and previously reported to be present in this patient in the absence of any other signs of CTD [1]. Other Rho GTPase-activating proteins included in the microarray comprised ARHGAP4 (RhoGAP4), ARHGAP9, ARHGAP12, ARHGAP15, ARHGAP24 (transcript variants 1 and 2), ARHGAP28, ARHGAP29, ARHGAP41 (oligophrenin-1, OPHN1), ARGAP47 (TAGAP), ARHGAP48 (FAM13A1), G3BP1, and SYDE1. However, none of these proteins showed significant binding of patient IgG (signal ranks 852–8462; median *Z*-factor: −114,057).

ARHGAP17 is a neuron-associated GTPase-activating protein expressed in the cerebellum, hippocampus and cerebral cortex [16], which is involved in regulating Ca(2+)-dependent exocytosis, presumably by catalyzing GTPase-activity and by inducing the reorganization of the cortical actin filaments [16]. Expression of ARHGAP17 in a pheochromocytoma-derived cell line resulted in strong inhibition of NGF-dependent neurite outgrowth [17]. Human Protein Atlas (HPA) data suggest strong expression, as demonstrated at the RNA level, also in oligodendrocytes (Fig. 1A). HPA and UniProt/SwissProt data—in line with data from the literature—support localization of ARHGAP17 to both the cytosol and the plasma membrane and tight junctions [18, 19]. The presence of ARHGAP17 in neurons and oligodendrocytes renders it conceivable that additional reactivity of ARHGAP26-IgG/anti-Ca-positive sera with ARHGAP17 could contribute to the pathogenesis of autoimmune encephalitis, and the degree of additional reactivity/cross-reactivity with ARHGAP17 (and ARHGAP10) might explain some of the observed differences between patients regarding disease severity, treatment response, lesion sites, or clinical presentation.

**Fig. 1:**
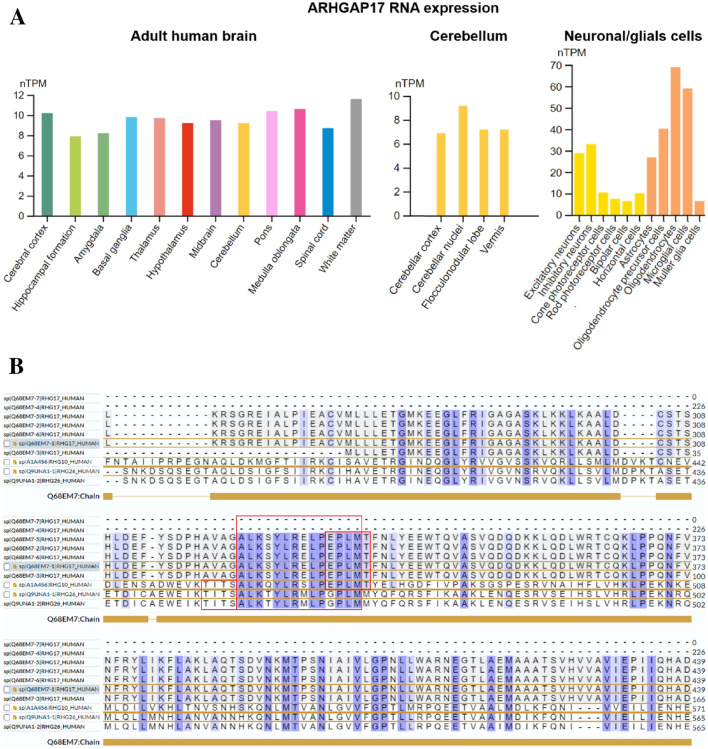
ARGAP17 expression in the human brain and sequence alignments of ARHGAP17, ARHGA26 and ARHGAP10 revealing a potential shared binding site. **A** ARHGAP17 expression levels in various subregions of the adult brain and cerebellum as well as in selected neuronal and glial cell types (modified images from the Human Protein Atlas image database [23]; https://www.proteinatlas.org; licensed under the Creative Commons Attribution-ShareAlike 3.0 International License). **B** Significant sequence homology of ARHGAP26, ARHGAP10 and ARHGAP17 within the RhoGAP domain (ARHGAP26: aa 383–568, ARHGAP10: aa 389–574, ARHGAP17: 252–442 aa) as detected using UniProt sequence data and ClustalO multiple sequence alignment tool (see Supplementary figure for additional information) [18, 24]. By contrast, no such significant sequence homology with ARHGAP26, ARHGA10 and ARHGAP17 in the RhoGAP domain exists in 12 other ARHGAPs studied in the same microarray experiment, all of which did not show significant binding of patient IgG (not shown); and homology only with the ALK_Y sequence of ARHGAP26, ARHGAP10 and ARHGAP17 (and the GALK_Y sequence of ARHGAP17) exists in the case of ARHGAP2 (N-chimerin), which yielded a weak signal in the same experiment (not shown). Together, this suggests that the RhoGAP domain could be the main binding site of the patient’s anti-ARHGAP IgG antibodies. Note that 2 (out of 7) isoforms of ARHGAP17 (Q68EM7-4 und Q68EM7-7) lack the region of interest

So far, systematic data on cross-reactivities are missing for most autoantibodies, although multiple reactivities have indeed been previously observed in a few other paraneoplastic neurological syndromes. For example, anti-Yo-positive sera bind to CDR2 (cerebellar degeneration-related 2; less commonly termed CDR62), CDR3 [20], a protein similar to CDR2, and, in 85%, to CDR2L (cerebellar degeneration-related 2-like) [2]. Similarly, anti-Ma-positive sera bind to paraneoplastic antigen Ma1 and paraneoplastic antigen Ma2 [21]. It may thus be worthwhile to evaluate possible co-reactivities against homologous proteins also in other types of autoimmune encephalitis.

Interestingly, all three Rho GTPase-activating antigens targeted by this patient’s serum IgG – ARHGAP26 [1], ARHGP10 [15], and ARHGAP17 –, activate, at least according to in vitro data, the GTPase Cdc42. It is therefore worth mentioning that in addition significant reactivity of this patient’s serum to the Rho GTPase binding/CDC42 effector proteins 3 and 2 (CDC42EP3, CDC42EP2) was observed (signal rank 54 and 172, respectively, of 9483 proteins; median signal intensities: 3374 and 1489; median *Z*-factors: 0.85 and 0.78).

For the sake of completeness, it should not go unmentioned that weak reactivity was found also with ARHGAP2 (N-chimerin, CHN1) in the same experiment (signal rank 249; median FU at 635 nm: 1166; *Z*-factor: 0.66). ARHGAP2 is a brain GTPase-activating protein (specific for Rac1 rather than Cdc42) with highest levels of expression in Purkinje cells in the cerebellum and in neurons of the hippocampus and cortex that has been implicated in pruning of dendritic spines and branches in response to synaptic activity [22]; suppression of ARHGAP2 expression was followed by increased process growth from the dendritic shaft and from spine heads [22].

Of note, substantial sequence homology exists between ARHGAP26, ARHGAP10 and ARHGAP17 within the RhoGAP domain (Fig. 1B) [18], which is not shared by those 12 ARHGAPs that did not show a significant reaction with the patient’s IgG in the same microarray experiment and only partly shared by ARHGAP2, which yielded only a weak signal (Supplementary Figure), rendering the RhoGAP domain a potential binding site recognized by the patient’s anti-ARHGAP antibodies in all three ARHGAP proteins. The assumption of cross-reactivity is also strongly corroborated by the fact that we could demonstrate a significant correlation of ARHGAP10-IgG/anti-Ca2 titres with ARHGAP26-IgG/anti-Ca titres (*p* < 0.0001) in our previous study [15]. Moreover, co-reactivity with ARHGAP10 was observed in almost all ARHGAP26-IgG/anti-Ca-positive patients in that study [15], which renders a coincidence highly unlikely. Taken together, this strongly favors cross-reactivity of a single antibody entity recognizing a shared epitope present in all three ARHGAPs in our patient over the presence of three distinct antibody entities with different epitope specificities.

Further studies are now warranted to define the frequency of anti-ARHGAP17 antibodies among patients with autoimmune encephalitis, in particular among those with autoimmunity to ARHGAP26, and the immunopathogenetic and, potentially, clinical and therapeutic significance of this new reactivity. So far, autoimmune encephalitis syndromes have been mostly understood to be caused by a reaction against single, distinct antigens. However, our findings suggest that anti-neural autoimmunity might be much more complex than previously thought and can involve cross-reactivity to a multitude of structurally related but functionally diverse proteins with differential expression patterns throughout the CNS. This could explain some of the variety in clinical manifestation observed in patients with one and the same autoantibody-defined disorder. Our data provide a strong rationale for future studies investigating such cross-reactivities also in other autoimmune diseases of the CNS and may alter our understanding of the pathophysiology of autoimmune encephalitis.

## Supplementary Information

Below is the link to the electronic supplementary material.

Supplementary file1 (DOCX 1371 KB)

## Abbreviations

ACA: Autoimmune cerebellar ataxia
ARHGAP: Rho GTPase-activating protein
CDC42EP: CDC42 effector protein
CDR: Cerebellar degeneration-related protein
CDR2L: Cerebellar degeneration-related 2-like protein
CHN1: N-chimerin
IgG: Immunoglobulin G
FU: Fluorescence units
OPHN1L: Oligophrenin-like protein 1
RICH-1: RhoGAP interacting with CIP4 homologs protein 1
