# Supplementary material for: Rho GTPase-activating protein 17 (ARHGAP17) as additional autoimmune target in ARHGAP26-IgG/anti-Ca autoantibody-associated autoimmune encephalitis
Source: J Neurol. 2022 Nov 4;270(3):1776–80. doi: 10.1007/s00415-022-11417-z (PMC9971044; doi:10.1007/s00415-022-11417-z)
Supplement: Supplementary file 1 — Supplementary file1 (DOCX 1371 KB) [file 415_2022_11417_MOESM1_ESM.docx]

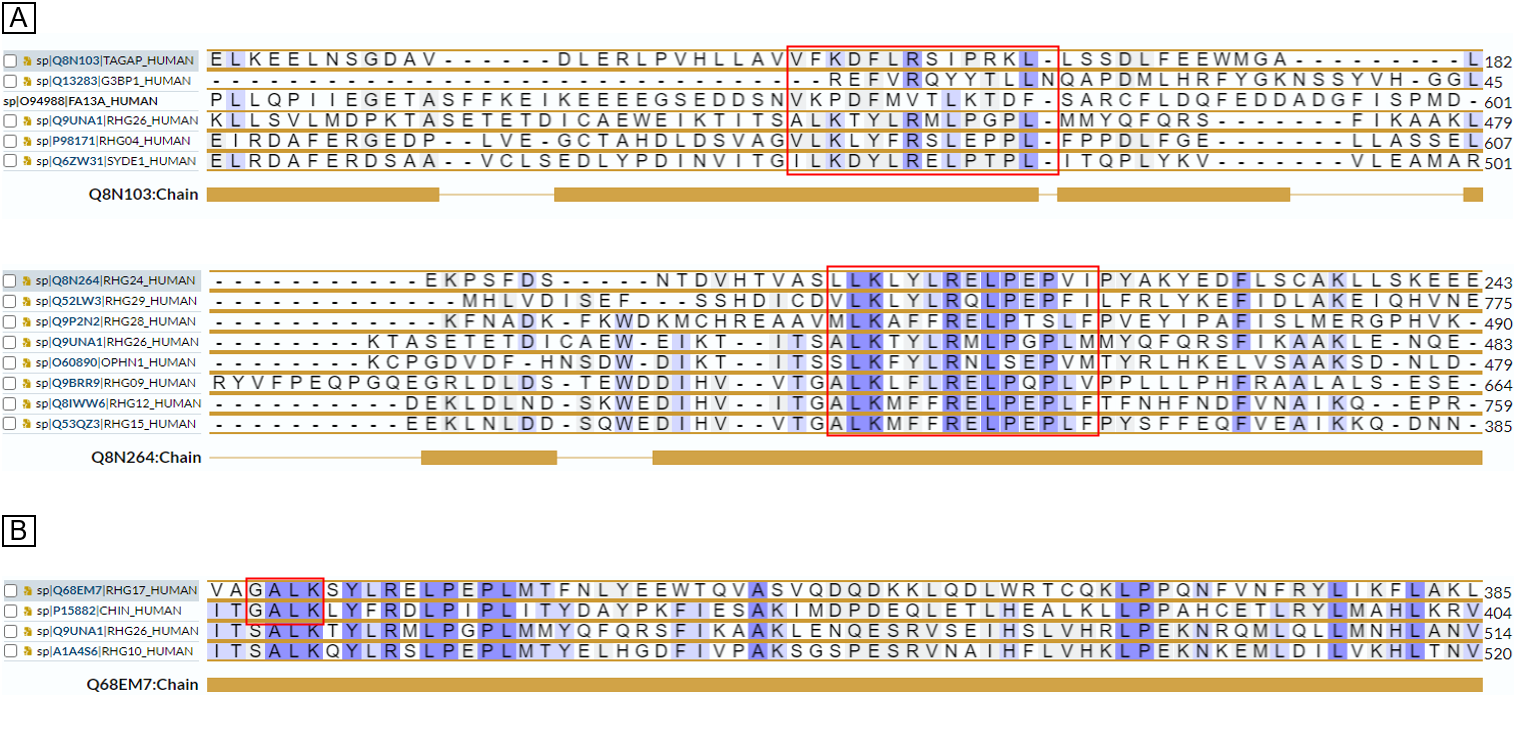


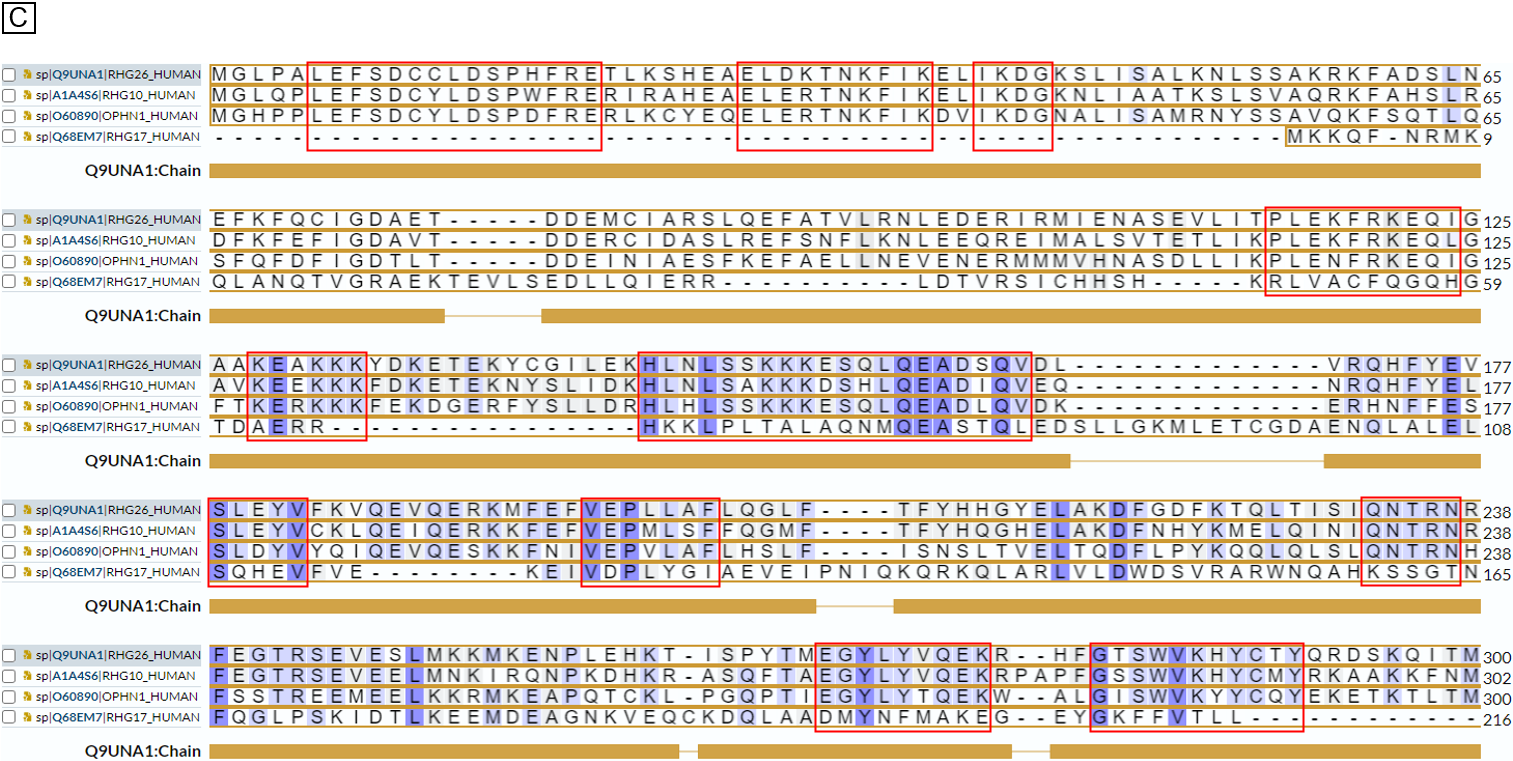


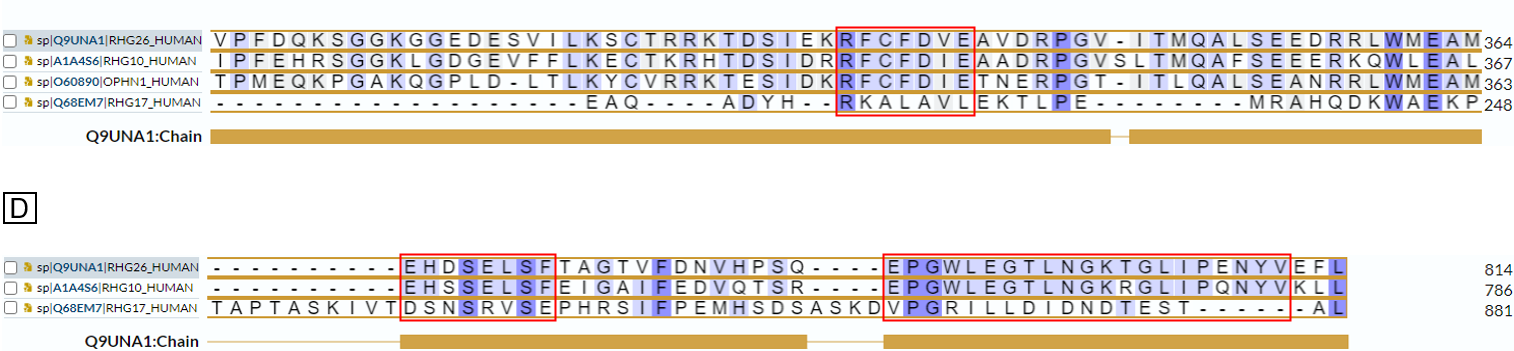


Supplementary figure. Selected alignments of the canonical sequences of ARHGAP26 and other ARHGAPs present on the same microarray chip. (A) The ALK_YLR and the LP_PLM sequence (red frames), which is shared by all of the three ARHGAPs recognized by the patient’s serum IgG (ARHGAP26, ARHGAP10, ARHGAP17), as depicted in Figure 2, is absent in 12 other ARHGAPs on the same microarray chip, all of which did not significantly react with the patient’s serum IgG in the same microarray experiment (ARHGAP4, ARHGAP9, ARHGAP12, ARHGAP15, ARHGAP24, ARHGAP28, ARHGAP29, ARHGAP41, ARGAP47, ARHGAP48, G3BP1, and SYDE1), suggesting the RhoGAP domain may contain the dominant epitope. (B) ARHGAP2 (N-chimerin), which yielded a weak reaction, is the only other ARHGAP studied that shares the RhoGAP domain ALK_Y sequence with ARHGAP26, ARHGAP10 and ARHGAP17 (and contains the GALK_Y sequence of ARHGAP17), which makes that domain a possible shared binding site for all four antibodies. (C) Although ARHGAP26 shares the longest homologous sequence within the RhoGAP domain (TITS_ALK_YLR_LP_PLM) with ARHGAP10 of all ARHGAPs studied, it should not go unmentioned that ARHGAP26 and ARHGAP10 share significant homology also in the N-terminal region. However, there is substantial N-terminal homology also with oligophrenin-1, which was not detected by the patient’s serum IgG in the same microarray experiment, and no such N-terminal homology exists with ARHGAP17, which yielded the second strongest signal, rendering it unlikely that the patient’s antibodies bind (at least predominantly) to this region of ARHGAP26/10. (D) Finally, significant homology between ARHGAP26 and ARHGAP10 exists within the C-terminal SH domain, but not between ARHGAP26 and ARHGAP17 (Supplementary Figure 1D). While we cannot fully exclude that subsets of the patient’s serum IgG repertoire recognize epitopes present only on one or two of these three ARHGAPs, the lack of sequence homology between ARHGAP26 and ARHGAP17 in the SH domain rather favours the RhoGAP sequences shared by all three ARHGAPs (Figure 1) as the most likely binding site over the SH domain.
